# The C–F bond as a conformational tool in organic and biological chemistry

**DOI:** 10.3762/bjoc.6.38

**Published:** 2010-04-20

**Authors:** Luke Hunter

**Affiliations:** 1 School of Chemistry, The University of Sydney, NSW 2006, Australia

**Keywords:** conformation, functional molecules, organofluorine chemistry, stereochemistry, stereoelectronic effects

## Abstract

Organofluorine compounds are widely used in many different applications, ranging from pharmaceuticals and agrochemicals to advanced materials and polymers. It has been recognised for many years that fluorine substitution can confer useful molecular properties such as enhanced stability and hydrophobicity. Another impact of fluorine substitution is to influence the conformations of organic molecules. The stereoselective introduction of fluorine atoms can therefore be exploited as a conformational tool for the synthesis of shape-controlled functional molecules. This review will begin by describing some general aspects of the C–F bond and the various conformational effects associated with C–F bonds (i.e. dipole–dipole interactions, charge–dipole interactions and hyperconjugation). Examples of functional molecules that exploit these conformational effects will then be presented, drawing from a diverse range of molecules including pharmaceuticals, organocatalysts, liquid crystals and peptides.

## Review

### General aspects of the C–F bond

Fluorine is a small atom, with an atomic radius intermediate between that of hydrogen and oxygen (Table 1). The small size of fluorine means that it can be incorporated into an organic molecule as a replacement for hydrogen without dramatically affecting the overall molecular size. However, fluorine is the most electronegative element in the periodic table, consequently the C–F bond is highly polarised and in this sense it is a dramatic change from a C–H bond [1–2]. In the highly polarised C–F bond, the fluorine atom bears a partial negative charge and the carbon atom bears a partial positive charge, and these charges attract each other. Hence, the C–F bond has significant ionic character; it is a very short and strong bond. The fluorine atom has three lone pairs, but because of fluorine’s high electronegativity these lone pairs are tightly held by the nucleus and are therefore quite unreactive (fluorine is only a very weak H-bond acceptor, for example). Another consequence of the highly polarised nature of the C–F bond is a low-energy σ* antibonding orbital, which is located behind the carbon atom in the plane of the C–F bond. This vacant orbital can accept electron density from nearby electron-donating groups such as lone pairs or σ-bonds and the importance of this will be discussed in the next section. Overall, the C–F bond can be thought of as short, strong, polarised and unreactive.

**Table 1:** Properties of some common elements and of their bonds to carbon [2–3].

	H	**F**	O	N	C	Cl	Br

Van der Waals radius (Å)	1.20	**1.47**	1.52	1.55	1.70	1.75	1.85
Pauling electronegativity	2.1	**4.0**	3.5	3.0	2.5	3.2	2.8
Length of single bond to carbon (Å)	1.09	**1.40**	1.43	1.47	1.54	1.77	1.97
Strength of bond to carbon (kcal/mol)	98	**105**	84	70	83	77	66

### Conformational effects associated with C–F bonds

#### Dipole–dipole interactions

**Figure 1:**
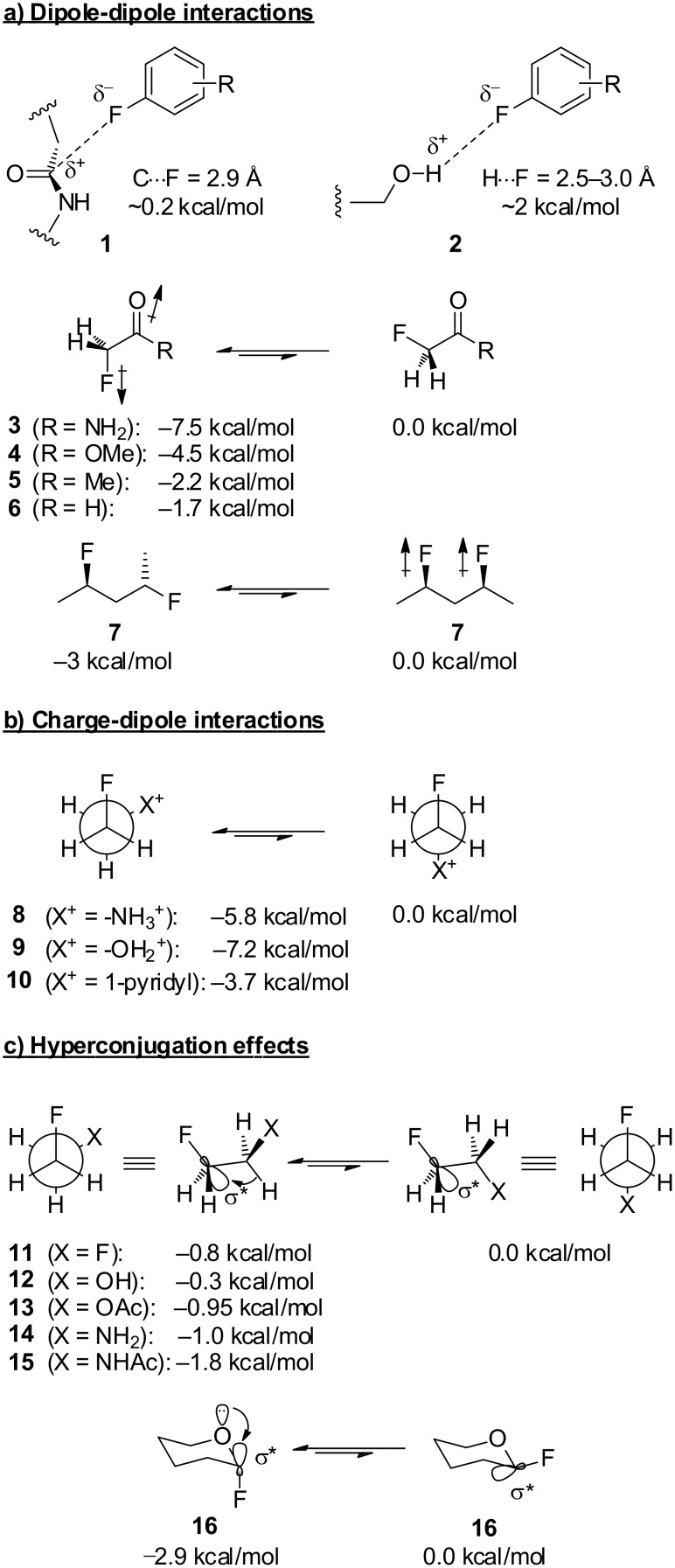
Conformational effects associated with C–F bonds.

We now have a picture of the C–F bond as a highly polarised unit containing a hard, partially negative fluorine atom. This picture suggests that the C–F bond should interact with its environment principally through electrostatic (dipole–dipole and charge–dipole) interactions. Such interactions can indeed be observed in an intermolecular sense, where, for example, fluorine-containing drug molecules can bind their receptor with the fluorine atom oriented towards a partial positive charge such as an amide carbon or an acidic hydrogen in a protein receptor (**1** and **2**, Figure 1a) [4–5]. However, it should be emphasised that such intermolecular electrostatic interactions are quite weak: for example, the C–F···H–O interaction (**2**) is at most one-quarter as strong as a “normal” hydrogen bond [2].

In contrast, electrostatic interactions can also occur within an organofluorine molecule and these can be substantially stronger. For example, in α-fluoroamides (e.g. **3**, Figure 1a) there is a strong preference for the C–F bond to align antiparallel to the C=O bond, a conformation in which the C–F dipole opposes the amide dipole. An analogous effect exists with other α-fluorocarbonyl compounds, but the effect decreases with the decreasing dipole moment of the carbonyl group (**4**–**6**, Figure 1a) [2].

As well as stabilising certain conformations, dipole–dipole interactions can also be responsible for destabilising other conformations. For example, in 1,3-difluoroalkanes (e.g. **7**, Figure 1a) there is an energetic penalty associated with the conformation in which the two C–F bonds are aligned parallel [6–7]. Molecules containing 1,3-*syn* fluorine substituents will therefore prefer to twist in order to avoid parallel 1,3-C–F dipoles. An alternative explanation for the 1,3-difluoro repulsion effect invokes a steric clash between the fluorine atoms, but since fluorine is a small atom, the dipole repulsion argument is more convincing.

#### Charge–dipole interactions

Electrostatic interactions associated with the C–F bond become more pronounced when a neighbouring group bears a formal charge [8]. For example, in the 2-fluoroethylammonium ion (**8**) and protonated 2-fluoroethanol (**9**) (Figure 1b), the *gauche* conformers are strongly preferred because these bring the (partially negative) fluorine atoms close to the formally positively-charged oxygen or nitrogen [9]. It is possible to envisage an intramolecular hydrogen bond helping to stabilise the *gauche* conformers of **8** and **9**, but the *gauche* preference is also maintained in systems such as **10** (Figure 1b) which cannot accommodate a hydrogen bond [10], confirming that the charge–dipole interaction is more important than any weak H-bonding in these systems.

#### Hyperconjugation effects

Consider the well-studied molecule 1,2-difluoroethane (**11**, Figure 1c). There are two possible staggered conformers, with the fluorine atoms either *gauche* or *anti*. NMR and molecular modelling studies have shown that the *gauche* conformer is lower in energy, which is perhaps a surprising result since the fluorine atoms might reasonably be expected to repel each other. What effect overrides the difluoro repulsion and stabilises the *gauche* conformer?

There is a vacant low-energy σ* antibonding orbital associated with each C–F bond (Figure 1c). In the *gauche* conformer of **11**, both σ*_CF_ orbitals are aligned with adjacent C–H bonds, which can donate electron density into the σ*_CF_ orbitals in a process known as hyperconjugation [1–2]. Feeding electron density into an antibonding orbital in this way is equivalent to partially breaking the bond, so when hyperconjugation occurs the C–F bonds of **11** become longer and less covalent in character. However the bonds are still strong because the fluorine atoms have now become even more negative, so they are more strongly attracted to the partially positive carbon atoms. Overall, hyperconjugation is a stabilising effect and thus will lower the energy of the *gauche* conformer of **11**. In contrast, in the *anti* conformer of **11** each σ*_CF_ orbital is now aligned with an adjacent C–F bond, which is highly polarised and less electron releasing than a C–H bond and hence hyperconjugation does not occur.

The *gauche* effect is only a subtle conformational influence compared with the dipole–dipole and charge–dipole interactions described earlier. Nevertheless, the *gauche* effect is very general and applies in many other systems in addition to 1,2-difluoroalkanes. For example, compounds containing F–C–C–O and F–C–C–N also experience this effect (**12**–**15**, Figure 1c) [9,11–13]. In general, more electronegative substituents give rise to stronger *gauche* effects. It should be noted that there are other explanations for the *gauche* effect in addition to the hyperconjugation argument presented above. For example, the “bent bond” theory [11] is an alternative explanation for the *gauche* preference of compounds **11**–**15** (Figure 1c). However, the hyperconjugation argument is more widely cited today [2] and will be exclusively quoted in this review.

The examples of hyperconjugation presented thus far (**11**–**15**, Figure 1c) all feature σ-bonds as the electron-donating groups. However, hyperconjugation can also occur with other electron donors such as lone pairs [1,14] or π-systems [15]. In each case, conformations which align the electron-donating group with the σ*_CF_ orbital will be favoured (e.g. **16**, Figure 1c).

In summary, fluorine atoms influence the conformation of organic molecules through dipole–dipole interactions, charge–dipole interactions and hyperconjugation effects. All of these influences can be rationalised by considering that the C–F bond is short, strong and highly polarised. The remainder of this review will focus on examples of *shape-controlled functional molecules* that exploit the C–F bond as a conformational tool.

### Bioactive small molecules

Despite being the most abundant halogen in the Earth’s crust, fluorine is almost completely absent from natural products chemistry [16]. However, in contrast to the paucity of fluorinated molecules in nature, there are many *synthetic* (non-natural) organofluorine compounds with valuable biological activity. Of these, an interesting subset exploit the C–F bond specifically as a conformational tool and some examples of such molecules are examined below.

#### Fluorinated pharmaceuticals

**Figure 2:**
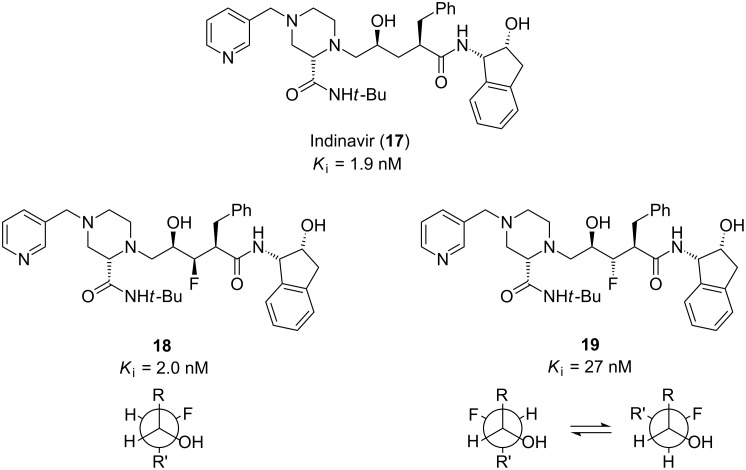
HIV protease inhibitor Indinavir (**17**) and fluorinated analogues **18** and **19**. In analogue **18** the *gauche* effect reinforces the active zigzag conformation, whereas in analogue **19** the *gauche* effect competes against the zigzag conformation resulting in conformational disorder.

A drug will bind its protein target with maximal affinity if it is pre-organised into the correct conformation prior to binding and this can be achieved in certain cases by judiciously incorporating fluorine atoms into the drug [4,17]. This concept is illustrated in structure–activity relationship studies of Indinavir (**17**, Figure 2), an HIV protease inhibitor developed by Merck. It is a functionalised pseudopeptide containing a central hydroxyethylene moiety in place of a scissile peptide bond. X-ray crystallography shows that **17** binds to HIV protease with its central carbon chain in an extended zigzag conformation [18]. To further investigate this binding mode, the fluorinated Indinavir analogues **18** and **19** were synthesised (Figure 2) [19]. Analogue **18** was shown to be equipotent with Indinavir (**17**), whereas the diastereomeric fluorinated analogue **19** was 14-fold less potent. The difference in potency between the fluorinated analogues can be attributed to the F–C–C–O *gauche* effect, which either reinforces (**18**) or destabilises (**19**) the bioactive extended chain conformation.

**Figure 3:**
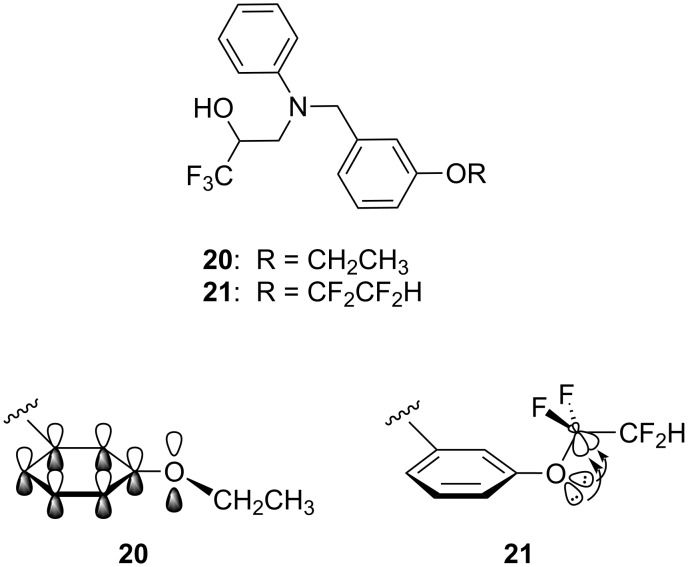
Cholesteryl ester transfer protein inhibitors **20** and **21**. In the fluorinated analogue **21**, n_O_→σ*_CF_ hyperconjugation leads to an out-of-plane orientation of the fluoroalkyl sidechain, resulting in improved binding affinity.

Another conformational effect of fluorine substitution is revealed in compounds **20** and **21** (Figure 3). These molecules are inhibitors of cholesteryl ester transfer protein, and are therefore of potential value in the treatment of coronary heart disease [20]. Alkoxyphenyl substituents (such as the ethoxy group of **20**) are known to align in the plane of the aryl ring (Figure 3). This is perhaps a surprising result given the additional steric demand of the in-plane conformation, but it can be rationalised by considering that the ether oxygen is sp^2^ hybridised [4] which allows its p-orbital to enter into conjugation with the aryl π-system. In contrast, the ether oxygen of the fluorinated analogue **21** is sp^3^ hybridised, which allows the two lone pairs to donate electron density into the two σ*_CF_ antibonding orbitals. As a result there is less conjugation between the oxygen lone pairs and the aryl π-system, so there is nothing to counteract the steric demand of an in-plane conformation, and thus the fluoroalkyl ether of **21** prefers an orthogonal orientation. In the case of inhibitor **21**, the orthogonal orientation of the fluorinated sidechain results in more efficient binding to the target protein, translating into an 8-fold increase in potency relative to the non-fluorinated analogue **20**.

**Figure 4:**
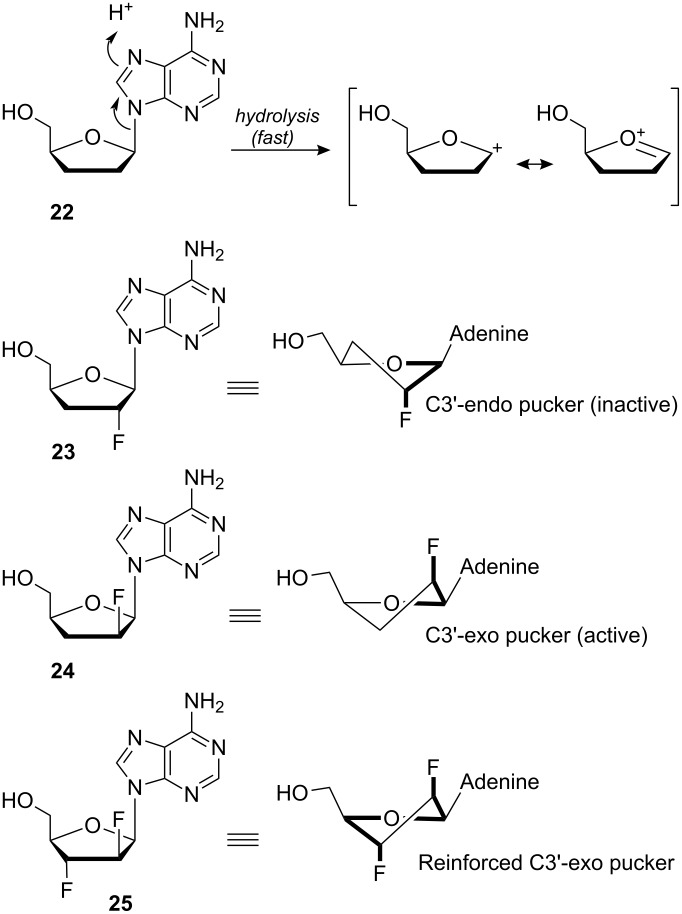
HIV reverse transcriptase inhibitor **22** and acid-stable fluorinated analogues **23**–**25**. The F–C–C–O *gauche* effect influences the ring conformations of **23**–**25**.

There has been a large amount of research into fluorinated nucleoside analogues as potential treatments for cancer and viral infection [21–22]. Fluorine is an obvious choice for incorporating into sugar-modified nucleoside analogues, since fluorine can be considered a reasonable mimic of either a hydrogen atom or a hydroxyl group. Fluorine atoms have a strong influence on both the electronic and the conformational properties of the sugar moiety, and these effects are illustrated in a series of antiviral compounds **22–25** (Figure 4) [23]. Dideoxy adenosine (**22**) is an inhibitor of HIV reverse transcriptase, but its clinical use is hampered by low hydrolytic stability. This problem can be overcome by incorporating a fluorine atom in the C2′ position (**23** and **24**, Figure 4). The enhanced acid-stability of **23** and **24** is due to the fluorine atom inductively destabilising the glycosyl carbonium ion hydrolytic intermediate. Interestingly however, fluorinated isomer **23** is inactive against HIV reverse transcriptase, whereas the diastereomeric compound **24** maintains the potency of the parent compound **22**. This result can be explained by the effect of the fluorine atoms on the molecular conformations of **23** and **24** [24]. In isomer **23**, the fluorine atom aligns *gauche* to the ring oxygen, resulting in a C3′-*endo* ring pucker which is not recognised by HIV reverse transcriptase [24–25]. By contrast, in isomer **24** the fluorine once again aligns *gauche* to the ring oxygen, but this leads to a C3′-*exo* ring pucker which is known to be optimal for biological activity. This effect can be explored further by incorporating a second fluorine atom at the C3′ position (**25**, Figure 4). If the C3′ stereochemistry is appropriate, the C3′-*exo* ring pucker can be further reinforced, with both fluorines aligned *gauche* to the ring oxygen (note that a potential difluoro *gauche* effect is overridden in this case) [24,26].

**Figure 5:**
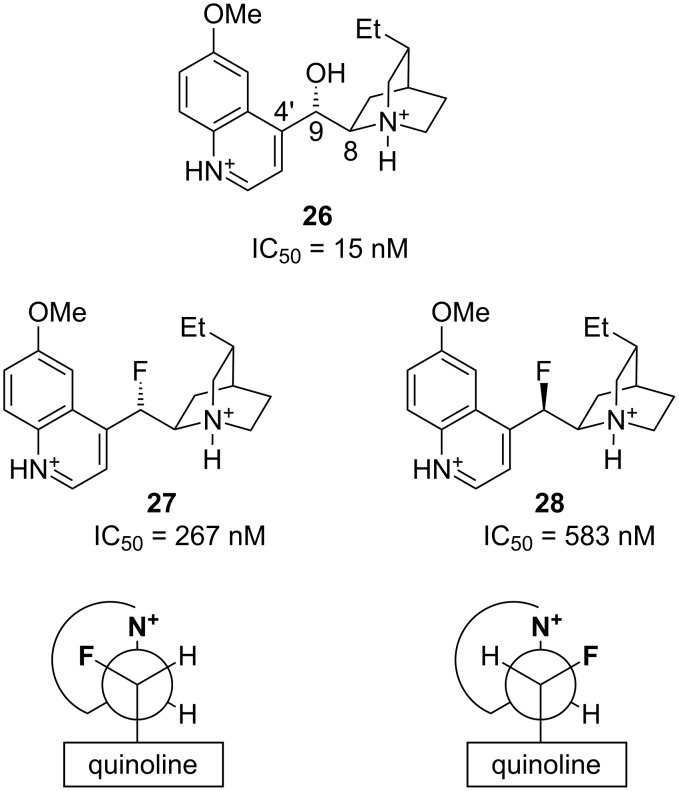
Dihydroquinidine (**26**) and fluorinated analogues **27** and **28**. Newman projections along the C9–C8 bonds of **27** and **28** show the proposed bioactive conformation.

Dihydroquinidine (**26**, Figure 5) is a highly active anti-malarial alkaloid. It has conformational degrees of freedom about the C9–C4′ and C8–C9 bonds, and some information about the bioactive conformation of **26** can be obtained from the fluorinated analogues **27** and **28** (Figure 5) [27]. Although there is a reduction in potency upon replacing the hydroxyl group of **26** with a fluorine atom, the fluorinated analogues **27** and **28** nevertheless maintain anti-malarial activity in the nanomolar range. Interestingly, **27** and **28** have quite similar activities (only a two-fold difference in potency). A possible interpretation of this result is that the bioactive conformation is as illustrated in Figure 5, since both isomers **27** and **28** benefit from a *gauche* F–C–C–N^+^ alignment in this conformation. Such an analysis is reinforced by NMR data which clearly show that **27** and **28** adopt the illustrated conformations about the C8–C9 bond in methanol solution.

#### Biological probes

**Figure 6:**
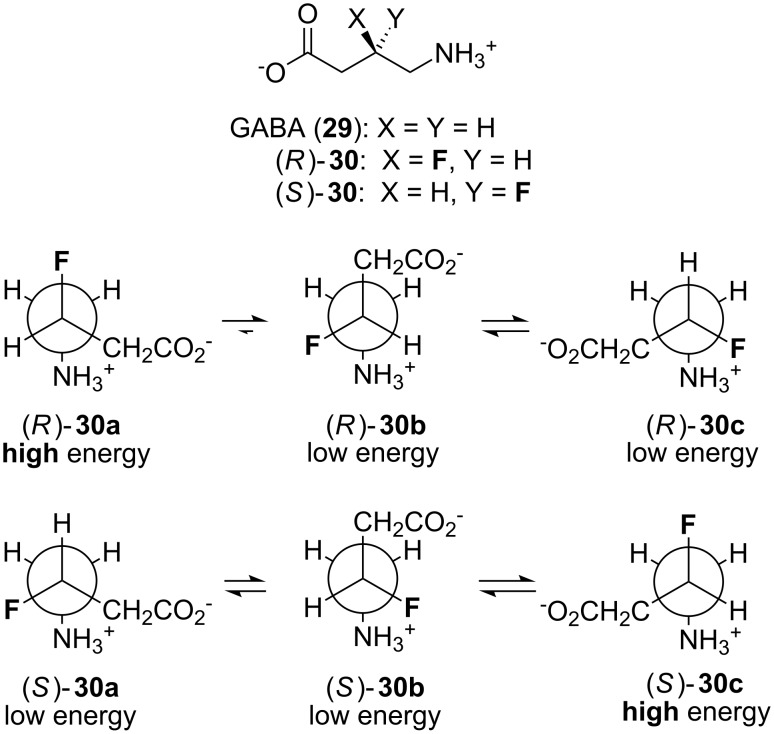
The neurotransmitter GABA (**29**) and fluorinated analogues (*R*)-**30** and (*S*)-**30**. Newman projections of (*R*)-**30** and (*S*)-**30** along the C3–C4 bond show the three possible staggered conformations (“**a**”, “**b**” and “**c**”).

γ-Aminobutyric acid (GABA, **29**, Figure 6) is an important neurotransmitter molecule. It is quite a flexible molecule, with 3 rotatable C–C bonds. GABA (**29**) binds to several different proteins, including various (GABA)-gated ion channels and the metabolising enzyme GABA-aminotransferase. In order to rationally design drugs that are specific for individual GABA-binding proteins, it is necessary to know the conformation that the flexible molecule GABA adopts when binding that particular protein. One method to gain this information is to investigate the fluorinated GABA analogues (*R*)-**30** and (*S*)-**30** (Figure 6) [28]. Each of (*R*)-**30** and (*S*)-**30** can adopt three possible staggered conformations about the C3–C4 bond, but because of a charge–dipole attraction between the fluorine and nitrogen atoms, these staggered conformations have different energies. Comparison of the binding affinities of (*R*)-**30** and (*S*)-**30** for a particular protein can therefore give information on the binding conformation of the natural ligand. For example, (*R*)-**30** and (*S*)-**30** are found to bind with equal affinity to the GABA_A_ synaptic receptor [28]. This suggests that the extended conformer (“**b**” in Figure 6) is the relevant binding mode since both (*R*)-**30** and (*S*)-**30** benefit from a *gauche* F–C–C–N^+^ alignment in this conformation, and therefore have approximately equal energies. In contrast, (*R*)-**30** is found to bind with more than 10-fold higher affinity than (*S*)-**30** to the metabolising enzyme GABA-aminotransferase [29]. This suggests that a bent conformer (“**c**” in Figure 6) is the relevant binding mode in this case, since (*R*)-**30** benefits from a *gauche* F–C–C–N^+^ alignment in conformer “**c**” whereas (*S*)-**30** does not.

**Figure 7:**
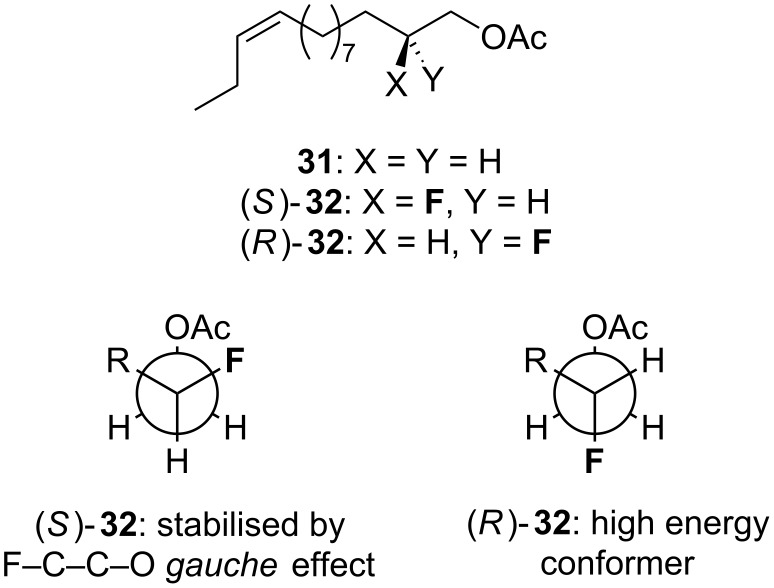
The insect pheromone **31** and fluorinated analogues (*S*)-**32** and (*R*)-**32**. The proposed bioactive conformation is shown in Newman projections.

In a similar vein, some information about the bioactive conformation of the insect pheromone **31** may be obtained by investigating the fluorinated analogues (*R*)-**32** and (*S*)-**32** (Figure 7) [30]. When (*R*)-**32** and (*S*)-**32** are compared in their ability to attract the relevant insect (the European corn borer, *Ostrinia nubilalis*), (*S*)-**32** is reported to possess similar biological activity to the parent non-fluorinated pheremone **31**, whereas (*R*)-**32** is inactive. This would suggest the bioactive conformation shown in Figure 7. However, this interpretation is speculative since the biological assay data is only preliminary, and the *gauche* effect in this system is relatively subtle (~1 kcal/mol).

**Figure 8:**
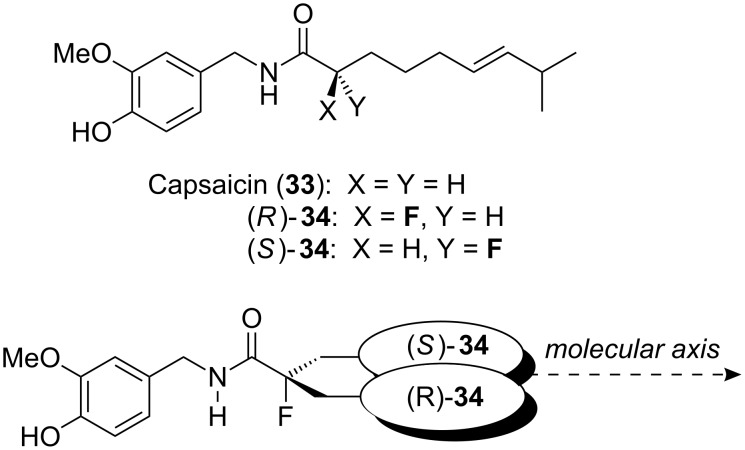
Capsaicin (**33**) and fluorinated analogues (*R*)-**34** and (*S*)-**34**.

Capsaicin (**33**, Figure 8) is a vanilloid natural product responsible for the pungency of chilli peppers. Its natural production is thought to protect the chilli pepper from predatory mammals. Capsaicin (**33**) binds to the pain receptor TRPV1, a non-selective cation channel that also responds to heat and acidic pH. Somewhat counterintuitively, capsaicin has been used for many years as a traditional medicine for the treatment of pain and there is considerable interest today in the production of capsaicin analogues as new analgesics. However, the binding mode of capsaicin (**33**) to the receptor TRPV1 is not known in full detail. The fluorinated analogues (*R*)-**34** and (*S*)-**34** (Figure 8) provide valuable information [31]. Due to the α-fluoroamide effect, the two enantiomers are expected to project the alkyl chain in different directions from the molecular axis, so the relative binding efficiency of (*R*)-**34** and (*S*)-**34** should inform on the binding conformation of natural capsaicin (**33**). It emerges that both enantiomers bind TRPV1 with similar affinity to capsaicin itself and this suggests that the alkyl chain projects roughly along the molecular axis when bound to TRPV1 since both enantiomers can approximate this conformation equally well [31]. This interpretation is in agreement with a previous study which made inferences from X-ray crystallography of a related receptor [32].

### Organocatalysts

So far we have seen that the C–F bond can be a valuable tool for medicinal chemists seeking to control the molecular conformation of drugs and bioprobes. This section will show that the C–F bond is also emerging as a useful tool in the field of catalysis. Recent reports have shown that organocatalysts can be conformationally “fine-tuned” by fluorine substitution for improved activity and selectivity.

**Figure 9:**
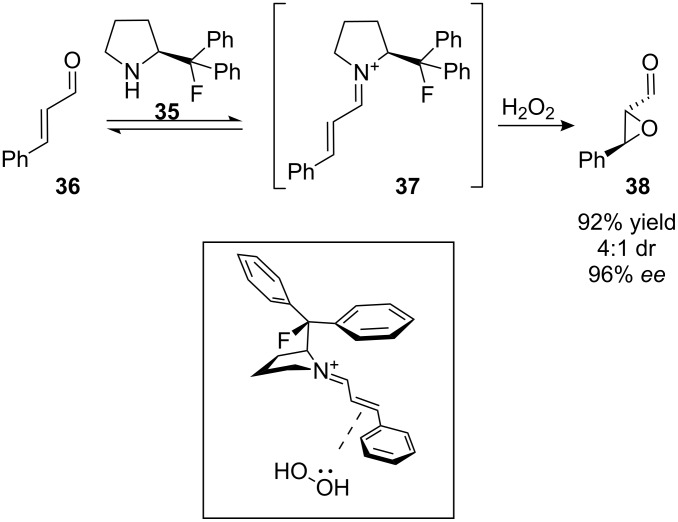
Asymmetric epoxidation reaction catalysed by pyrrolidine **35**. Inset: the geometry of the activated iminium ion intermediate **37** is stabilised by a *gauche* F–C–C–N^+^ alignment.

Pyrrolidine **35** (Figure 9) is a highly selective catalyst for the epoxidation of α,β-unsaturated aldehydes (e.g. **36**) [33]. In the first step of the reaction, aldehyde **36** and pyrrolidine **35** react together to form the iminium ion **37**. This has a LUMO-lowering effect (analogous to Lewis-acid activation of **36**) which makes **37** more reactive towards nucleophiles [34]. In intermediate **37**, the fluorine atom aligns *gauche* to the positively-charged nitrogen atom (Figure 9, inset), resulting in a phenyl group shielding the top (*re*) face of the alkene. Hydrogen peroxide consequently attacks from the bottom (*si*) face, leading to epoxide **38** with high enantioselectivity. In a control experiment, the related organocatalyst 2-(diphenylmethyl)pyrrolidine (containing a hydrogen atom instead of the fluorine atom of **35**) also catalyses the same reaction but with lower enantioselectivity suggesting that the fluorine atom of **35** helps to rigidify the activated intermediate and thereby enhances selectivity.

**Figure 10:**
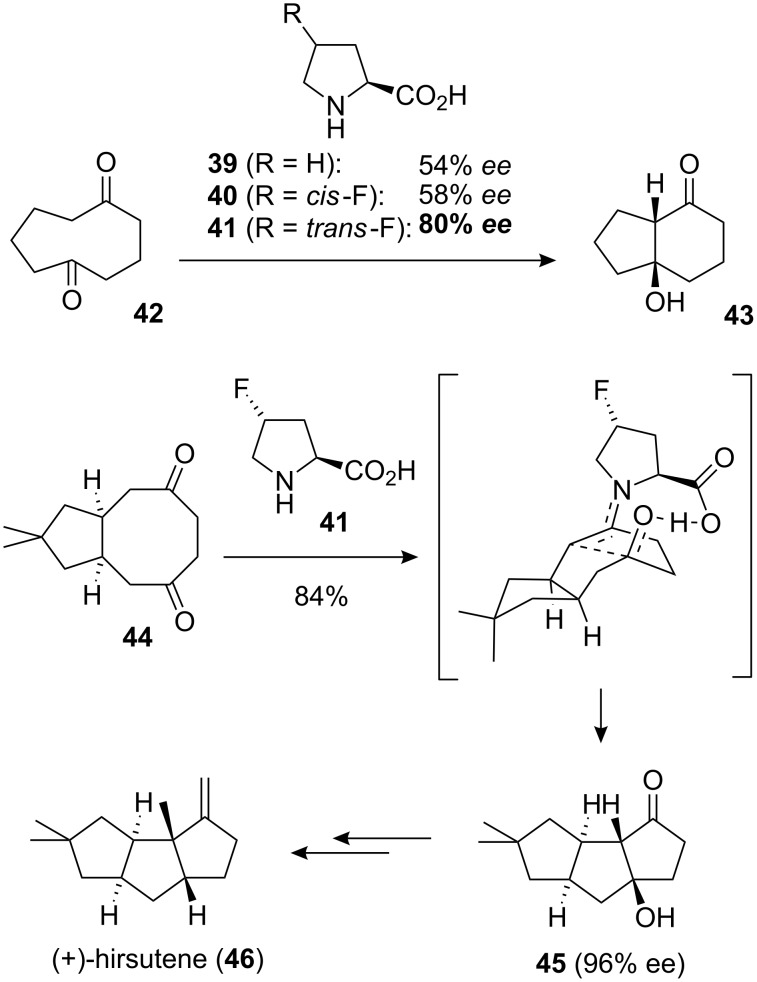
The asymmetric transannular aldol reaction catalysed by *trans*-4-fluoroproline (**41**), and its application to the total synthesis of (+)-hirsutene (**46**).

Another fluorinated organocatalyst has recently featured in the first example of an asymmetric transannular aldol reaction (Figure 10) [35]. (*S*)-proline (**39**) is able to catalyse this reaction with moderate enantioselectivity and a similar result is observed with *cis*-4-fluoroproline (**40**). However, a notable improvement in enantioselectivity is obtained with the diastereoisomeric catalyst *trans*-4-fluoroproline (**40**). The authors of this study report that further work to elucidate this fluorine effect is ongoing. Fluorine atoms are known to influence the conformation of pyrrolidine rings through the F–C–C–N *gauche* effect (see this review’s section on collagen for a further discussion of this effect in the context of fluorinated peptides). It is interesting to speculate whether a Cγ-*exo* proline ring shape, reinforced by the F–C–C–N *gauche* effect, could be partly responsible for the high enantioselectivity of catalyst **41**. As an illustration of the importance of this work, catalyst **41** has already been put to good use in a total synthesis of the natural product (+)-hirsutine (**46**, Figure 10), with the key transannular aldol reaction (**44**→**45**) proceeding in high yield and with impressive enantioselectivity [35].

**Figure 11:**
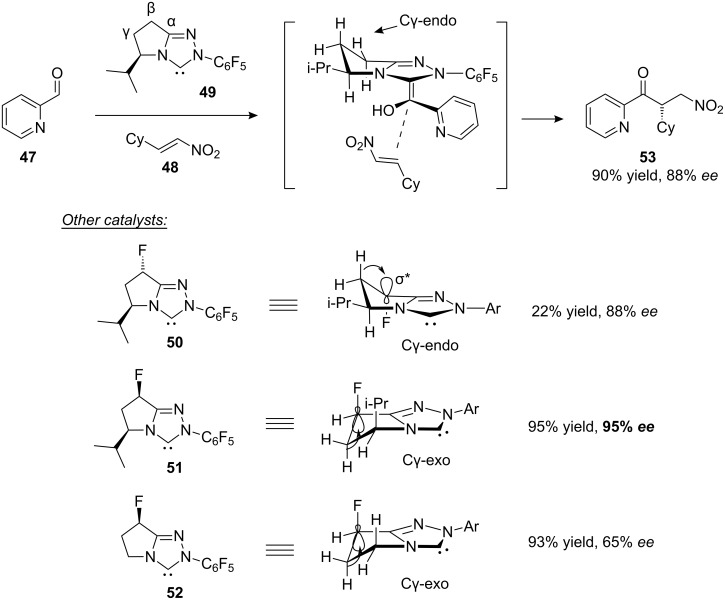
The asymmetric Stetter reaction catalysed by chiral NHC catalysts **49**–**52**. The ring conformations of **50**–**52** are influenced by σ_CH_→σ*_CF_ hyperconjugation. Cy = cyclohexyl.

Fluorine-substituted organocatalysts are also useful in the asymmetric Stetter reaction (Figure 11) [36]. *N*-Heterocyclic carbene **49** was identified as a promising first-generation catalyst for the Stetter reaction between aryl aldehydes (e.g. **47**) and nitroalkenes (e.g. **48**). Superficially, it seems that the bulky isopropyl group of **49** is solely responsible for the enantioselectivity of this reaction. However, the shape of the bicyclic ring system might also play a role and this idea can be explored by comparing catalyst **49** with the fluorinated analogues **50**–**52**. The parent catalyst **49** adopts a Cγ-*endo* ring conformation, which is favoured because of the pseudoequatorial orientation of the bulky isopropyl group. In catalyst **50** the Cγ-*endo* conformation is maintained (this time reinforced by hyperconjugation) and the enantioselectivity of the reaction is unchanged. In contrast, catalyst **51** adopts a Cγ-*exo* conformation. This seems surprising because the bulky isopropyl group is now forced into a pseudoaxial position, but the steric clash is more than compensated for by hyperconjugation. Catalyst **51** is found to be significantly more enantioselective than **50**, suggesting that the Cγ-*exo* ring shape could be responsible for the improvement. Consistent with this, catalyst **52** is still capable of a reasonable level of asymmetric induction despite lacking the isopropyl group. The enantioselectivity of catalyst **52** is achieved solely through the Cγ-*exo* ring shape (assuming zero steric effects associated with the small fluorine atom). Overall, this work illustrates the great potential of using the C–F bond as a conformational tool in the development of new and improved organocatalysts.

### Multi-vicinal fluoroalkanes

**Figure 12:**
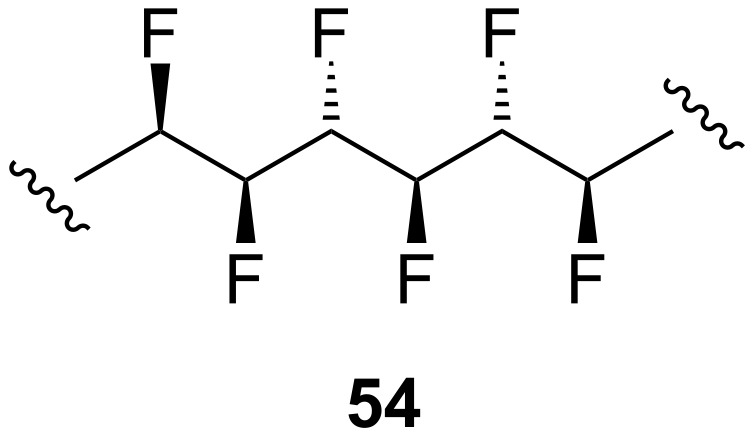
A multi-vicinal fluoroalkane.

We have already seen that in 1,2-difluoroethane (**11**, Figure 1c) the two vicinal C–F bonds align *gauche* to one another. What happens if there is a longer carbon chain containing several vicinal fluorine atoms? This gives rise to a new type of compound termed a “multi-vicinal fluoroalkane” (e.g. **54**, Figure 12), which is conceptually intermediate between alkanes and perfluoroalkanes [37]. Multi-vicinal fluoroalkanes are interesting systems for studying stereoelectronic effects such as the *gauche* effect and they also have potential applications in materials science, for example, as novel liquid crystals.

**Figure 13:**
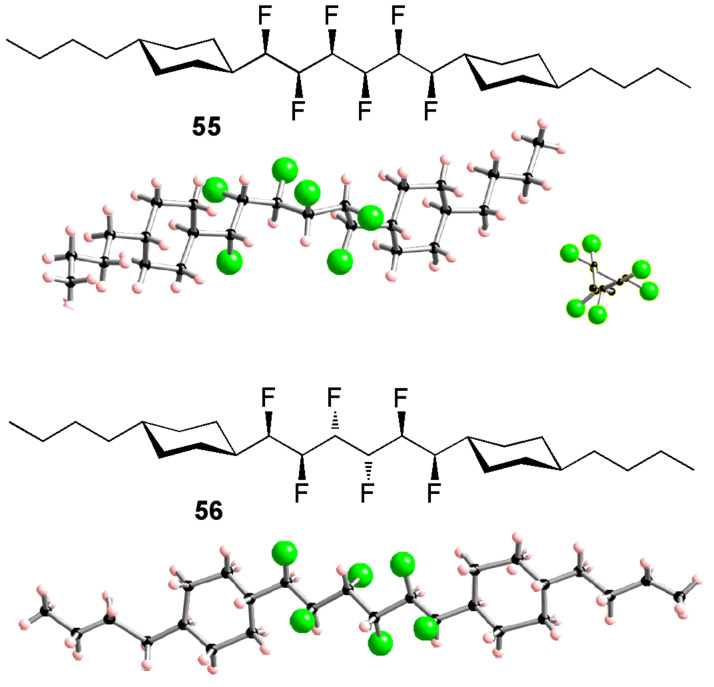
X-ray crystal structures of diastereoisomeric multi-vicinal fluoroalkanes **55** and **56**. The different conformations can be explained by (i) the avoidance of 1,3-difluoro repulsion and (ii) a preference for 1,2-difluoro *gauche* alignments.

A distinguishing feature of compounds such as **54** is their stereochemical complexity. It is necessary to control these stereocentres during synthesis so that the conformational properties of different diastereoisomers can be compared. This has been explored with compounds containing up to six vicinal fluorines [37–39] and it emerges that the conformations of these compounds are governed by two main considerations: parallel 1,3-C–F bonds are avoided, and *gauche* 1,2-C–F bonds are favoured. For example, consider the all-*syn* hexafluoroalkane **55** (Figure 13) [39]. This molecule cannot adopt a zigzag conformation because this would incur multiple 1,3-difluoro repulsions. Instead, **55** adopts a helical shape in which each pair of vicinal fluorines is aligned *gauche* but no 1,3-difluoro repulsion is present. In contrast, the diastereoisomeric compound **56** *does* adopt the zigzag conformation (Figure 13). This affords three out of a possible five 1,2-difluoro *gauche* alignments, while the different stereochemistry of the molecule prevents 1,3-difluoro repulsion from occurring.

**Figure 14:**
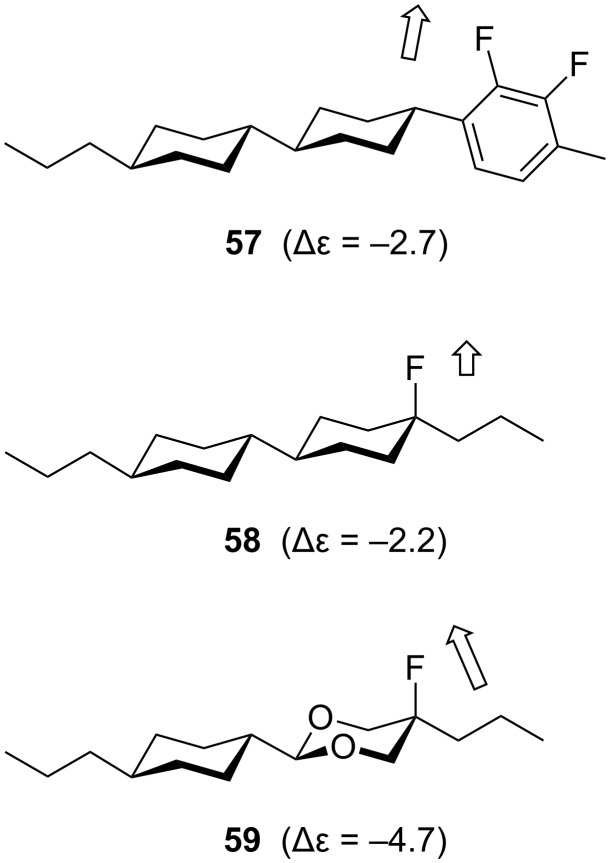
Examples of fluorinated liquid crystal molecules. Arrows indicate the orientation of the molecular dipole moments, which are quantified in the negative dielectric anisotropy values, Δε.

Knowledge of the conformational behaviour of multi-vicinal fluoroalkanes has informed the design of novel liquid crystals. A liquid crystal is a fluid phase in which there is some orientational ordering of the molecules. Liquid crystal display (LCD) technology requires rod-shaped molecules that have a dipole moment perpendicular to the long axis of the molecule, and this is often achieved by incorporating fluorinated subunits into the liquid crystal molecule (Figure 14) [40]. In most cases (e.g. **57** and **58**), the fluorine atoms act not as conformational control elements but simply as polar substituents. However, note that in the more sophisticated compound **59**, the ring oxygens also contribute to the dipole moment in addition to reinforcing the molecular conformation with two F–C–C–O *gauche* alignments [41].

**Figure 15:**
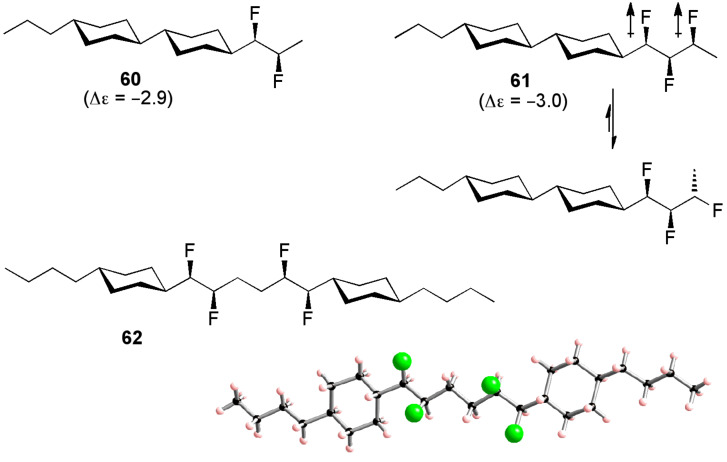
Di-, tri- and tetra-fluoro liquid crystal molecules **60**–**62**.

With a developing knowledge of the behaviour of multi-vicinal fluoroalkanes it has been possible to develop new liquid crystals containing several fluorine atoms, in which the fluorine atoms affect the molecular conformation as well as the molecular dipole moment. The difluoro compound **60** (Figure 15) can be viewed as a conceptual progression from the axially fluorinated liquid crystal **58**. NMR and modelling data show that the fluoroalkyl chain of **60** adopts a zigzag conformation in which the two C–F bonds are aligned *gauche* to one another [42]. Hence, both fluorine atoms are presented on the same face of the molecule, resulting in a substantial molecular dipole moment as measured in the large negative dielectric anisotropy value (Δε). This system can be extended to incorporate a third vicinal fluorine atom (**61**, Figure 15). Disappointingly however, the trifluoro analogue **61** seems to offer no improvement over the difluoro analogue **60** (almost identical values of Δε). This is because the conformation of **61** is affected by 1,3-difluoro repulsion. The fluoroalkyl chain of compound **61** cannot adopt the zigzag conformation because of this repulsion effect and hence the three fluorine atoms are not all presented on the same face of the molecule. This problem is overcome in the next-generation compound **62** (Figure 15) [39]. X-ray crystallography reveals that the fluoroalkyl chain of **62** adopts the desired zigzag conformation, which maximises the number of fluorine *gauche* alignments, with the insulating ethyl spacer preventing 1,3-difluoro repulsion. Interestingly, the X-ray structure of **62** reveals a slight twisting distortion about the molecular axis, possibly reflecting strain associated with a very high dipole moment caused by the orientation of all four fluorine atoms on the same face of the molecule. Overall, this work illustrates that a basic knowledge of the conformational preferences of multi-vicinal fluoroalkanes can have a valuable bearing on the design of functional materials.

### Peptides and proteins

Some of the most notable examples of exploiting the C–F bond as a conformational tool come from the world of peptides and proteins. The presence of amide functional groups in the peptide backbone provides a good opportunity to exploit the α-fluoroamide effect and the F–C–C–N *gauche* effect [43]. The concept of controlling peptide conformation using fluorine atoms is exciting because the conformation of a peptide critically affects its biological activity and consequently, there are many potential applications in medicinal chemistry and biotechnology.

#### Collagen

**Figure 16:**
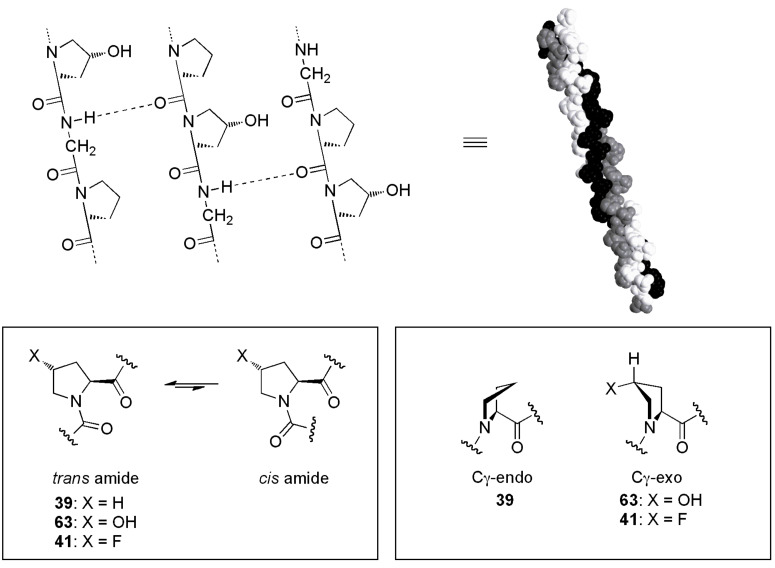
Collagen mimics of general formula (Pro-Yaa-Gly)_10_ where Yaa is either 4(*R*)-hydroxyproline (**63**) or 4(*R*)-fluoroproline (**41**). The fluorinated isomer is more stable, due to an increased preference for the *trans* amide bond and the Cγ-*exo* pyrrolidine ring pucker. The illustrated collagen triple helix structure is from PDB code 1CAG [45].

Collagen is the most abundant protein in animals. It is a structural protein responsible for the tensile strength of connective tissue. Collagen fibrils consist of a tight bundle of three parallel protein strands wound into a triple helix (Figure 16). Each protein strand is made of ~300 repeats of the sequence Xaa-Yaa-Gly, where Xaa is often proline (**39**) and Yaa is often 4(*R*)-hydroxyproline (**63**). The triple helix is partly held together by backbone hydrogen bonds and for many years it was thought that the hydroxyl groups of the 4(*R*)-hydroxyproline residues (**63**) contributed to the stability of collagen by providing extra hydrogen bonding. However, this theory was thrown into doubt when a collagen mimic was synthesised in which the 4(*R*)-hydroxyproline residues (**63**) were replaced with 4(*R*)-fluoroproline (**41**) [44]. Despite being unable to participate in interstrand hydrogen bonding, the 4(*R*)-fluoroproline residues were found to greatly increase the stability of the collagen triple helix. How could this be?

It emerges that rather than hydrogen bonding, the source of stability derives from conformational changes imparted by the fluorine substituent of **41** (Figure 16). For most peptide bonds, the *trans* conformation is strongly preferred and indeed an all-*trans* arrangement is required for the collagen strands to assemble into the triple helix. However, peptide bonds adjacent to proline residues have only a very slight *trans* preference, meaning that the *cis* isomer is also significantly populated in solution. In 4(*R*)-fluoroproline (**41**), the electronegative fluorine atom exerts an inductive “pull” which lowers the C(O)–N bond order [46]. This reduces the energy barrier to *cis*/*trans* isomerisation, allowing the peptide strand to pre-organise into the required all-*trans* conformation and thereby facilitating triple helix formation. More importantly, the fluorine substituent also affects the conformation of the proline ring (Figure 16). In unsubstituted proline residues **39**, the pyrrolidine moiety adopts a Cγ-*endo* ring pucker. In contrast, 4(*R*)-fluoroproline (**41**) exhibits a Cγ-*exo* pucker which is stabilised by a fluorine-amide *gauche* alignment [47]. There are several consequences of this, including further stabilising the *trans* amide through subtle mechanisms [48–49]. Crucially, the Cγ-*exo* pucker also means that the C–F bond is projected in such a way that it aligns antiparallel to three proximal C=O dipoles in the triple helix [47]. Thus, the fluorinated collagen mimic reveals that it is dipole–dipole interaction rather than hydrogen bonding that gives collagen its great stability.

#### Opioid receptor-binding peptides

**Figure 17:**
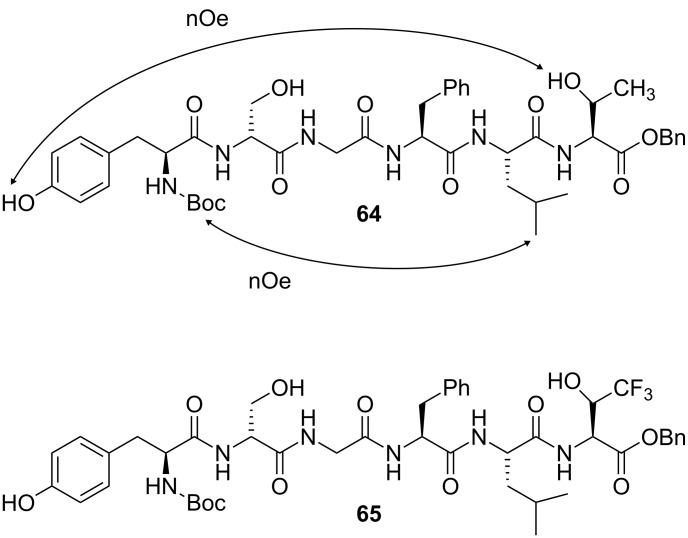
Enkephalin-related peptide **64** and the fluorinated analogue **65**. The electron-withdrawing trifluoromethyl group of **65** disrupts a key hydrogen bond, leading to a different conformation as determined by NOESY experiments.

The hexapeptide Tyr-D-Ser-Gly-Phe-Leu-Thr, known as the enkephalin-related peptide, binds to the δ-opioid receptor. Opioid receptor-binding peptides are of interest because of their biological roles in analgesia as well as in respiratory, gastrointestinal and cardiovascular functions [50]. However, their mechanism of action is difficult to elucidate, partly because these linear peptides are conformationally flexible. In order to gain information about the bioactive conformation, fluorine chemistry can be used to modify the peptides’ conformational behaviour. For example, there is an interesting contrast between the enkephalin-related peptide derivative **64** and its fluorinated analogue **65** (Figure 17) [51–52]. The NOESY spectrum of peptide **64** reveals long-range through-space interactions, suggesting a folded conformation possibly reinforced by a Tyr-OH···Thr-OH hydrogen bond. In contrast, analogue **65** contains an electron-withdrawing trifluoromethyl group, which lowers the H-bond acceptor ability of the adjacent hydroxyl group. The NOESY spectrum of **65** reveals no long-range interactions, suggesting that the crucial Tyr–Thr hydrogen bond is disrupted and that a linear peptide conformation is preferred.

#### Fluorinated β-peptides

**Figure 18:**
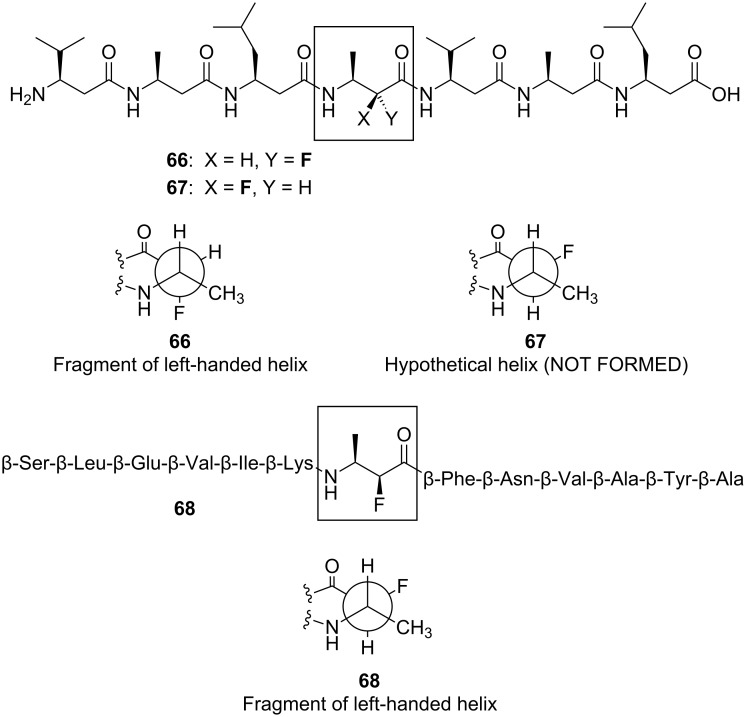
The C–F bond influences the conformation of β-peptides. β-Heptapeptide **66** adopts a helical conformation, reinforced by the α-fluoroamide effect and a fluorine-amide *gauche* alignment. In isomeric β-heptapeptide **67**, the helical conformation is disrupted by the fluorine atom. The disruptive effect of fluorine is overridden in the longer helix-forming β-tridecapeptide **68**.

β-Peptides are unnatural polymers composed of β-amino acids, which have an extra -CH_2_- group relative to natural α-amino acids (Figure 18). Despite the increased conformational freedom of β-peptides, they can nevertheless assemble into well-defined secondary structures such as helices, sheets and turns [53]. Certain β-peptidic structural motifs have been developed as effective mimics of biologically important α-peptides [54] and this holds great therapeutic promise because β-peptides are not recognised by hydrolase enzymes so have much longer half-lives in vivo [55].

One way to control the conformation of β-peptides is to incorporate fluorine atoms into the peptide backbone. This concept is elegantly illustrated by the diastereoisomeric β-peptides **66** and **67** (Figure 18) [56]. The β-amino acid sequence of **66** and **67** is known to promote the formation of a left-handed helix and this helical conformation can be either reinforced or destabilised by a fluorine substituent. In the case of β-peptide **66**, the fluorine atom aligns antiparallel to the adjacent C=O bond and *gauche* to the adjacent amide nitrogen, and this reinforces the helical conformation of the β-peptide. In contrast, the helical conformation of β-peptide **67** cannot accommodate these favourable alignments, so in this case the fluorine atom has a helix-breaking effect.

Interestingly, there is a limit to the conformational directing power of the C–F bond, as demonstrated by the longer β-tridecapeptide **68** (Figure 18) [57]. In this more extended system, the stronger propensity for helix formation overrides the conformational influence of the C–F bond, which is forced into a high-energy orientation orthogonal to the adjacent C=O bond. Nevertheless, taken together, the results with β-peptides (Figure 18) show that a single C–F bond can have a dramatic impact on peptide conformation.

#### Future directions

**Figure 19:**
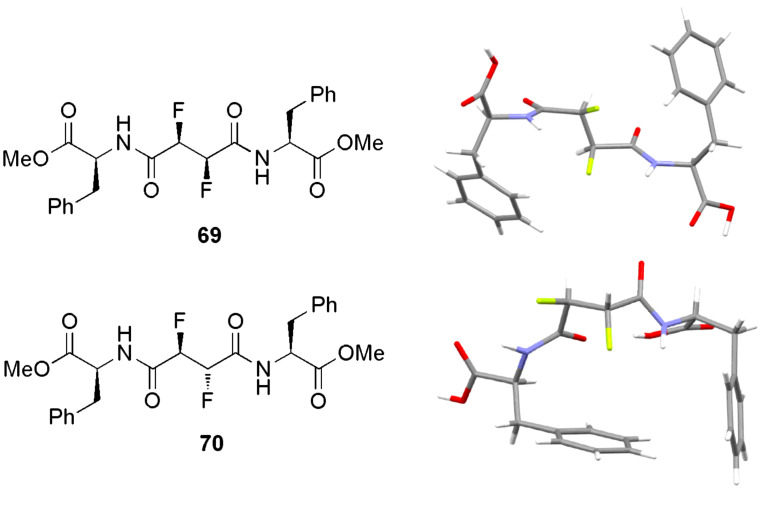
The conformations of pseudopeptides **69** and **70** are influenced by the α-fluoroamide effect and the fluorine *gauche* effect.

Recent results obtained with β-peptides illustrate that promising biological activity can be achieved with unnatural peptides [54]. This opens the door to a new area of research into more exotic amino acids containing several vicinal fluorine atoms. This would allow a greater variety of molecular shapes to be created, governed by the conformational rules known to operate in multi-vicinal fluoroalkanes in addition to the α-fluoroamide effect and the fluorine-amide *gauche* effect. Progress has been made towards this goal with the synthesis of pseudopeptides containing a difluorosuccinate core (**69** and **70**, Figure 19) [58–59]. In each of pseudopeptides **69** and **70**, the two fluorine atoms align antiparallel to the adjacent C=O bonds and *gauche* to one another, leading to different backbone conformations in the two diastereoisomers.

Building upon these promising results, a logical next step is to pursue the synthesis of non-symmetrical amino acids containing two or more vicinal fluorine atoms. Such fluorinated amino acids could be useful building blocks for the synthesis of shape-controlled bioactive pseudopeptides. Studies towards this goal are underway in the author’s laboratory, and details of these investigations will be reported in due course.

## Conclusion

The conformations of organofluorine compounds are influenced by a number of stereoelectronic effects associated with the C–F bond, including dipole–dipole interactions, charge–dipole interactions and hyperconjugation. Knowledge of these conformational effects allows the properties of functional molecules to be optimised through selective fluorination chemistry. This concept has been demonstrated in diverse areas including medicine, catalysis, materials science and biotechnology. It is hoped that the examples highlighted in this review have persuaded the reader of the great usefulness of the C–F bond as a conformational tool in organic and biological chemistry.

## References

[1] Kirsch P (2004). Modern Fluoroorganic Chemistry: Synthesis, Reactivity, Applications.

[2] O’Hagan D (2008). Chem Soc Rev.

[3] Smith M B, March J (2001). March’s Advanced Organic Chemistry: Reactions, Mechanisms, and Structure.

[4] Müller K, Faeh C, Diederich F (2007). Science.

[5] Böhm H-J, Banner D, Bendels S, Kansy M, Kuhn B, Müller K, Obst-Sander U, Stahl M (2004). ChemBioChem.

[6] Wu D, Tian A, Sun H (1998). J Phys Chem A.

[7] Hunter L, Slawin A M Z, Kirsch P, O’Hagan D (2007). Angew Chem, Int Ed.

[8] Sum A M, Lankin D C, Hardcastle K, Snyder J P (2005). Chem–Eur J.

[9] Briggs C R, Allen M J, O’Hagan D, Tozer D J, Slawin A M Z, Goeta A E, Howard J A K (2004). Org Biomol Chem.

[10] Gooseman N E J, O’Hagan D, Peach M J G, Slawin A M Z, Tozer D J, Young R J (2007). Angew Chem, Int Ed.

[11] Wiberg K B, Murcko M A, Laidig K E, MacDougall P J (1990). J Phys Chem.

[12] O’Hagan D, Bilton C, Howard J A K, Knight L, Tozer D J (2000). J Chem Soc, Perkin Trans 2.

[13] Briggs C R, O’Hagan D, Rzepa H S, Slawin A M Z (2004). J Fluorine Chem.

[14] Juaristi E, Cuevas G (1992). Tetrahedron.

[15] Tozer D J (1999). Chem Phys Lett.

[16] Deng H, O’Hagan D, Schaffrath C (2004). Nat Prod Rep.

[17] Purser S, Moore P R, Swallow S, Gouverneur V (2008). Chem Soc Rev.

[18] Chen Z, Li Y, Chen E, Hall D L, Darke P L, Culberson C, Shafer J, Kuo L C (1994). J Biol Chem.

[19] Myers A G, Barbay J K, Zhong B (2001). J Am Chem Soc.

[20] Massa M A, Spangler D P, Durley R C, Hickory B S, Connolly D T, Witherbee B J, Smith M E, Sikorski J A (2001). Bioorg Med Chem Lett.

[21] Pankiewicz K W (2000). Carbohydr Res.

[22] Meng W-D, Qing F-L (2006). Curr Top Med Chem.

[23] Marquez V E, Tseng C K-H, Mitsuya H, Aoki S, Kelley J A, Ford H, Roth J S, Broder S, Johns D G, Driscoll J S (1990). J Med Chem.

[24] Barchi J J, Karki R G, Nicklaus M C, Siddiqui M A, George C, Mikhailopulo I A, Marquez V E (2008). J Am Chem Soc.

[25] Van Roey P, Salerno J M, Chu C K, Schinazi R F (1989). Proc Natl Acad Sci U S A.

[26] Mikhailopulo I A, Pricota T I, Sivets G G, Altona C (2003). J Org Chem.

[27] Bucher C, Sparr C, Schweizer W B, Gilmour R (2009). Chem–Eur J.

[28] Deniau G, Slawin A M Z, Lebl T, Chorki F, Issberner J P, Van Mourik T, Heygate J M, Lambert J J, Etherington L-A, Sillar K T (2007). ChemBioChem.

[29] Clift M D, Ji H, Deniau G, O’Hagan D, Silverman R B (2007). Biochemistry.

[30] Khrimian A P, Oliver J E, Waters R M, Panicker S, Nicholson J M, Klun J A (1996). Tetrahedron: Asymmetry.

[31] Winkler M, Moraux T, Khairy H A, Scott R H, Slawin A M Z, O’Hagan D (2009). ChemBioChem.

[32] Jordt S-E, Julius D (2002). Cell.

[33] Sparr C, Schweizer W B, Senn H M, Gilmour R (2009). Angew Chem, Int Ed.

[34] MacMillan D W C (2008). Nature.

[35] Chandler C L, List B (2008). J Am Chem Soc.

[36] DiRocco D A, Oberg K M, Dalton D M, Rovis T (2009). J Am Chem Soc.

[37] Hunter L, O’Hagan D (2008). Org Biomol Chem.

[38] Farran D, Slawin A M Z, Kirsch P, O’Hagan D (2009). J Org Chem.

[39] Hunter L, Kirsch P, Slawin A M Z, O’Hagan D (2009). Angew Chem, Int Ed.

[40] Hird M (2007). Chem Soc Rev.

[41] Kirsch P, Hahn A, Fröhlich R, Haufe G (2006). Eur J Org Chem.

[42] Nicoletti M, Bremer M, Kirsch P, O’Hagan D (2007). Chem Commun.

[43] Briggs C R S, O’Hagan D, Howard J A K, Yufit D S (2003). J Fluorine Chem.

[44] Holmgren S K, Taylor K M, Bretscher L E, Raines R T (1998). Nature.

[45] Bella J, Eaton M, Brodsky B, Berman H M (1994). Science.

[46] Eberhardt E S, Panasik N, Raines R T (1996). J Am Chem Soc.

[47] Holmgren S K, Bretscher L E, Taylor K M, Raines R T (1999). Chem Biol.

[48] Bretscher L E, Jenkins C L, Taylor K M, DeRider M L, Raines R T (2001). J Am Chem Soc.

[49] Panasik N, Eberhardt E S, Edison A S, Powell D R, Raines R T (1994). Int J Pept Protein Res.

[50] Marcotte I, Separovic F, Auger M, Gagné S M (2004). Biophys J.

[51] Kitamoto T, Marubayashi S, Yamazaki T (2006). Chem Lett.

[52] Kitamoto T, Marubayashi S, Yamazaki T (2008). Tetrahedron.

[53] Seebach D, Hook D F, Glättli A (2006). Pept Sci.

[54] Seebach D, Gardiner J (2008). Acc Chem Res.

[55] Hook D F, Gessier F, Noti C, Kast P, Seebach D (2004). ChemBioChem.

[56] Mathad R I, Gessier F, Seebach D, Jaun B (2005). Helv Chim Acta.

[57] Mathad R I, Jaun B, Flögel O, Gardiner J, Löweneck M, Codée J D C, Seeberger P H, Seebach D (2007). Helv Chim Acta.

[58] Schüler M, O’Hagan D, Slawin A M Z (2005). Chem Commun.

[59] O’Hagan D, Rzepa H S, Schüler M, Slawin A M Z (2006). Beilstein J Org Chem.

